# Primary diffuse large B-cell lymphoma of the cranial vault with Trousseau syndrome: a case report

**DOI:** 10.1186/s13256-021-02979-z

**Published:** 2021-08-18

**Authors:** Tatsuya Uchida, Kenichi Amagasaki, Atsushi Hosono, Hiroshi Nakaguchi

**Affiliations:** Department of Neurosurgery, Mitsui Memorial Hospital, 1 Kandaizumicho, Chiyoda-ku, Tokyo, Japan

**Keywords:** Skull, Diffuse Large B-Cell Lymphoma, Cerebral stroke

## Abstract

**Background:**

It is extremely rare for primary non-Hodgkin’s lymphomas to occur singly in the cranial vault. One case diagnosed as primary diffuse large B-cell lymphoma is reported, initially misdiagnosed as metastatic skull tumor, complicated with Trousseau syndrome.

**Case description:**

The patient was a 60-year-old Japanese woman with no particular previous medical history. In a head computed tomography examination for vertigo, bone destructive skull tumor covering the right frontal, parietal, and temporal bones was incidentally discovered. As positron emission tomography indicated an abnormal accumulation in the large intestine and multiple cerebral infarctions suspicious of Trousseau syndrome were observed on magnetic resonance images, a metastatic skull tumor due to colorectal cancer was first considered. However, various tumor markers were negative, and colonoscopic biopsy indicated no colorectal abnormality. After pathological examination of the resected tumor, it was diagnosed as diffuse large B-cell lymphoma. The tumor affected muscles and skin but did not develop in the brain or the dura mater. As further general examination revealed no other abnormalities, we considered that it was primary diffuse large B-cell lymphoma in the cranial vault associated with Trousseau syndrome. Treatment with rituximab, cyclophosphamide, doxorubicin, vincristine, and prednisolone and high-dose methotrexate reduced the residual lesion; coagulation abnormalities, which are frequently associated with Trousseau syndrome, also improved.

**Conclusions:**

Skull tumors can result from a variety of malignancies, and their diagnosis may be complicated with Trousseau syndrome. However, even in cases of a single lesion in the cranial vault without invasion of the central nervous system, diffuse large B-cell lymphoma should be considered as a differential diagnosis.

## Introduction

Primary diffuse large B-cell lymphoma (DLBCL), a non-Hodgkin’s lymphoma, commonly occurs in the midline and deep white matter, such as the periphery of the lateral ventricle, basal ganglia, and cerebellum. Occurrence of DLBCL in the cranial vault is rare and accounts for 0.3–5.0% of all non-Hodgkin’s lymphoma [1–3]. It might often be difficult to diagnose initially owing to similarities with other skull tumors [4].

Trousseau syndrome is one of the paraneoplastic neurologic syndromes that causes neurological symptoms due to the remote effect of latent malignant tumors. Trousseau syndrome causes cerebral infarction due to hypercoagulation associated with malignant tumors [5].

One case diagnosed as primary diffuse large B-cell lymphoma (DLBCL) complicated with Trousseau syndrome is reported.

## Case report

**Fig. 1:**
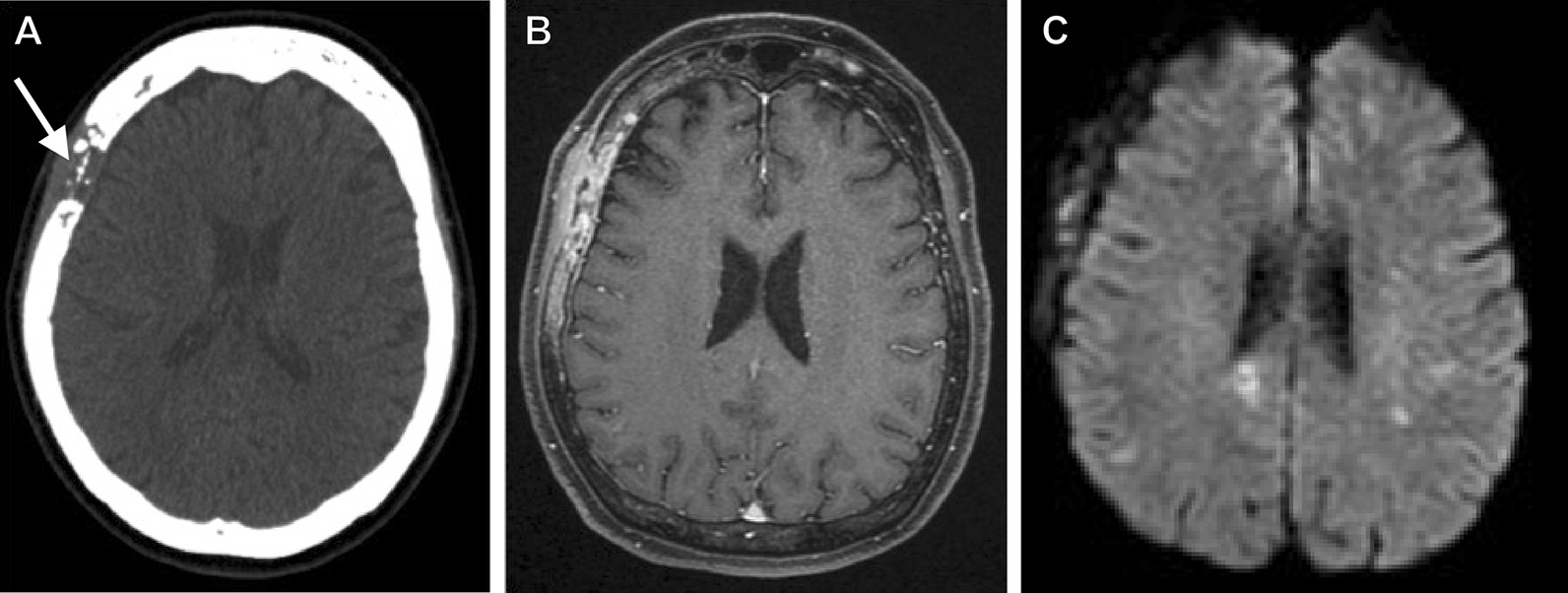
Preoperative images. **A** Head computed tomography (CT) showing bone destructive skull tumor spreading from the right frontal bone to the parietal and temporal bones (arrow). **B** Preoperative T1-weighted contrast-enhanced magnetic resonance imaging showing that the legion had invaded the surrounding subcutaneous tissue and temporal muscle. **C** Diffusion-weighted image showing new multiple cerebral infarctions in the bilateral hemispheres

A 60-year-old Japanese woman who had no immunodeficiency and an unremarkable medical history visited our hospital with sudden right facial paralysis and vertigo as the chief complaints. On neurological examination, she had no motor or sensory abnormalities other than facial paralysis and no cranial nerve symptoms. At that time, we detected a bone destructive skull tumor extending from the right frontal bone to the parietal and temporal bones on a head computed tomography (CT) scan (Fig. 1A). However, no obvious abnormalities were observed from visual inspection and palpation of the head.

**Fig. 2:**
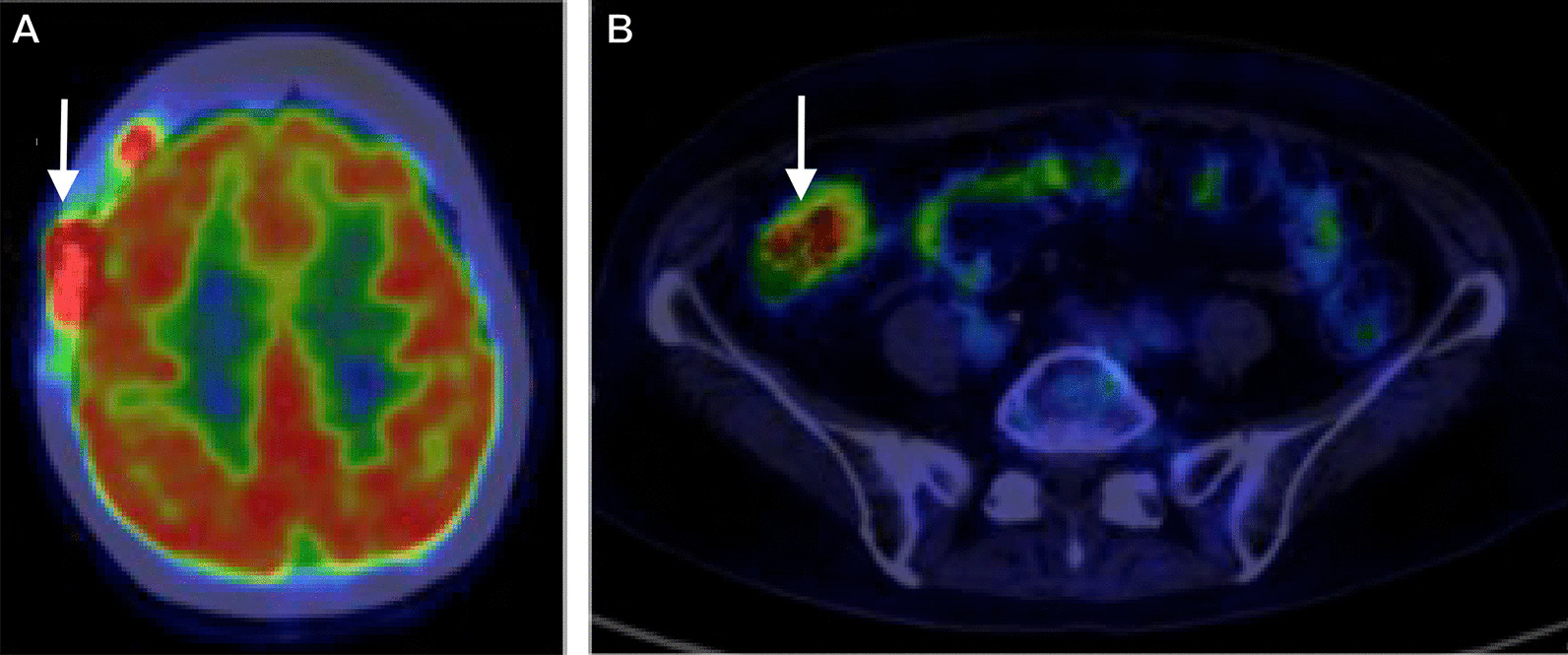
Positron emission tomography–CT images. **A** Abnormal accumulation consistent with the bone lesion (arrow). **B** Abnormal accumulation in the large intestine was confirmed (arrow)

**Fig. 3:**
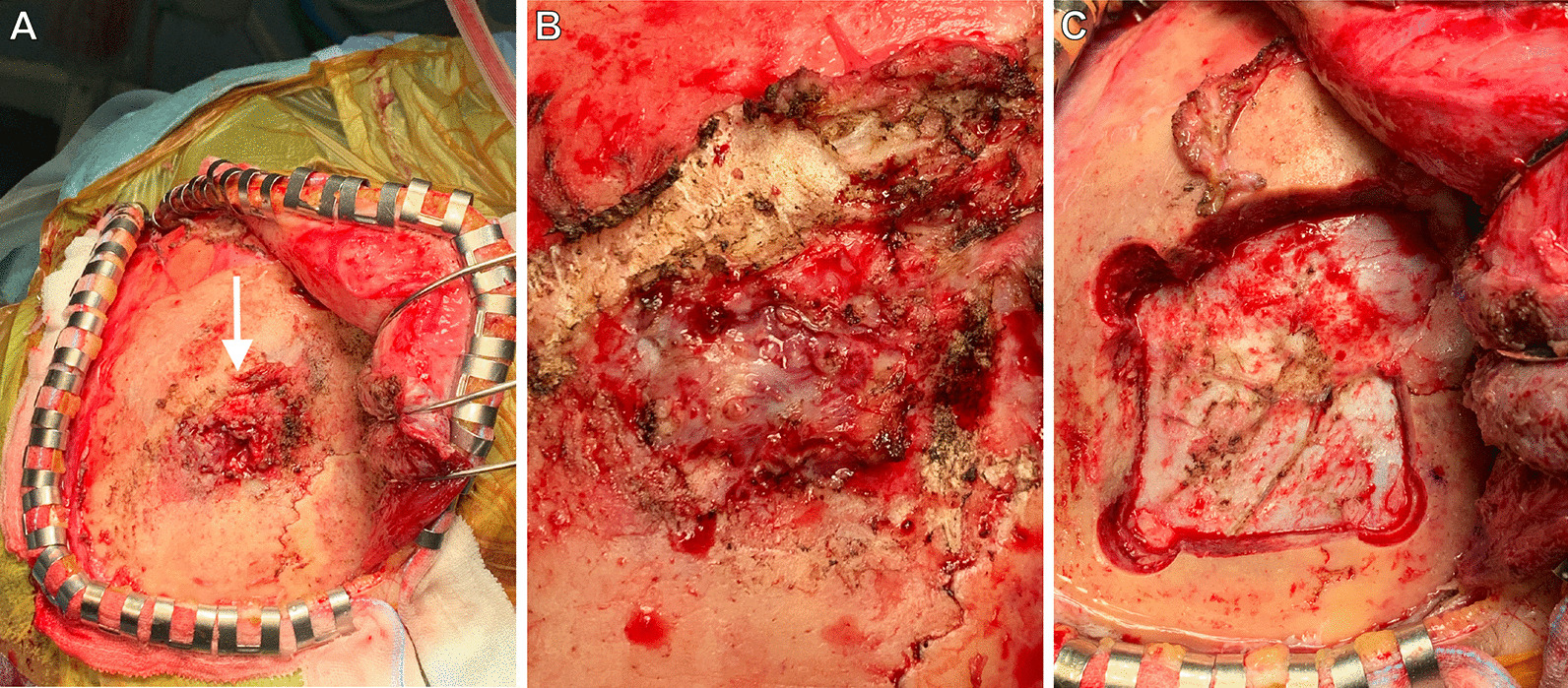
Intraoperative photographs. **A** Bone destruction was found mainly from frontal bone to parietal bone with the abundance of indurated tumor inside (arrow). **B** The tumor was hard and hemorrhagic. **C** The fractured bone and surrounding bone were removed, and it was confirmed that there was no infiltration into the dura mater

**Fig. 4:**
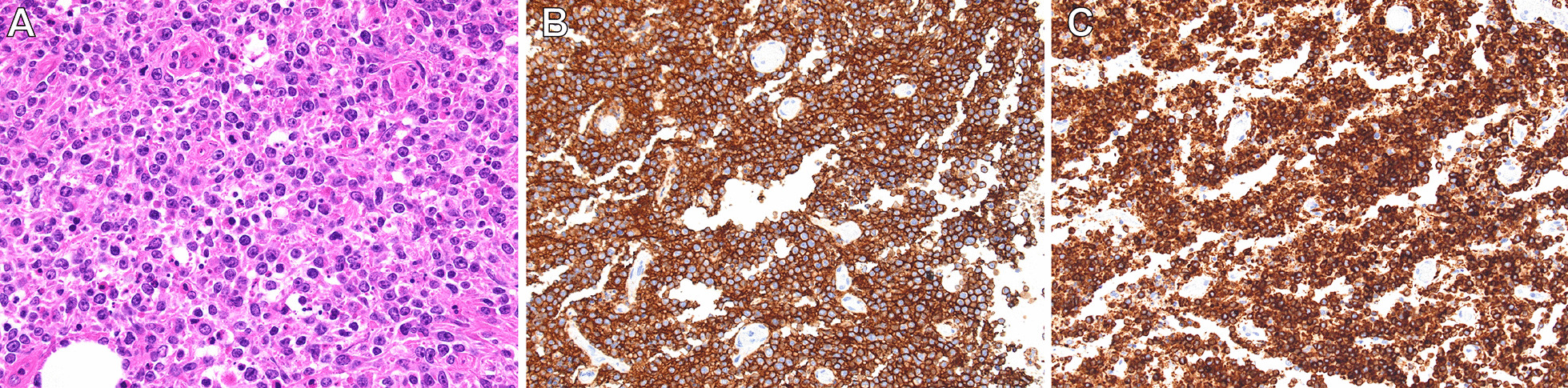
Histopathological findings of the resected skull tumor. **A** Large atypical cells invaded diffusely, and individual tumor cells with irregular nuclei were observed (hematoxylin and eosin stain, ×40). **B** Histological examination showing that the large tumor cells were immunoreactive for CD20 (immunohistochemistry, CD20, ×20). **C** Histological examination showing that the large tumor cells were immunoreactive for CD79a (immunohistochemistry, CD79, ×20)

Using contrast-enhanced magnetic resonance imaging (CEMRI), the lesion was confirmed to have invaded the surrounding subcutaneous tissue and temporal muscle (Fig. 1B), but it had not yet developed in the brain. Diffusion-weighted imaging revealed multiple acute ischemic lesions in bilateral hemispheres (Fig. 1C). Additionally, we confirmed abnormal accumulations consistent with the bone lesion (Fig. 2A) as well as in the large intestine (Fig. 2B) by positron emission tomography-CT (PET-CT). However, no abnormality was detected on colonoscopy, and all tumor markers—carcinoembryonic antigen, carbohydrate antigen 19-9, α-fetoprotein, soluble interleukin-2 receptor, protein induced by vitamin K absence or antagonist II, sialyl-Lewis X, cancer antigen 125, and β2 microglobulin—were negative. Contrast-enhanced CT did not reveal phlebothrombosis or any abnormal finding in the neck, chest, abdomen, and pelvis. The skull tumor was resected for confirming the diagnosis. When the bones were exposed in a wide area by performing a curved skin incision from the anterosuperior portion of the auricle to the midline, bone destruction was found mainly from the frontal bone to the parietal bone, with an abundance of indurated tumor inside (Fig. 3A). The tumor was highly vascularized with temporal muscle/subcutaneous invasion (Fig. 3B). We confirmed that there was no invasion to the dura mater by removing the destructed bone and the fractured pieces in the surrounding area (Fig. 3C). We covered the defective part of the bone with a plate and conducted coagulation for the invasion sites in the muscle. There were no complications due to surgery. Resection of the tumor was confirmed by postoperative MRI. Histopathologically, the resected lesion was diffusely invaded by large atypical cells with a high nucleo-cytoplasmic ratio; there were individual tumor cells with irregular nuclei as well as cells with distinctive nuclear bodies. Mitosis and necrosis were also observed (Fig. 4A). Immunohistochemical examination was positive for CD20/CD79a (Fig. 4B, C) and negative for CD3 in terms of atypical cells; the Ki-67 labeling index was approximately 90%. Based on these results, the tumor was identified as a DLBCL. Further examinations were conducted on the basis of the pathological results. Cerebrospinal fluid cytology examination and bone marrow biopsy were both negative for malignant cells. The blood sample was negative for viral markers, including HIV, with no abnormalities in blood counts and images. Coagulative markers indicated an increase in fibrin/fibrinogen degradation products (FDP) (70 μg mL), D-dimer (6.7 μg/mL), and thrombin–antithrombin III complex (TAT) (9.1 μg/L), but protein C and protein S levels were normal. Furthermore, various autoantibody, anticardiolipin antibody, and lupus anticoagulant tests were negative. Vascular stenosis was not observed on magnetic resonance angiogram and contrast-enhanced CT. There were no abnormalities in carotid ultrasonography and 24-hour electrocardiographic monitoring. Transesophageal echocardiography showed no nonbacterial thrombotic endocarditis findings and patent foramen ovale.

Based on all the test results, we confirmed the diagnosis as primary DLBCL of the cranial vault complicated with Trousseau syndrome. Trousseau syndrome was treated with continuous infusion of heparin during the perioperative period. On the third day after surgery, we switched the treatment to oral anticoagulant medication. After completing three courses of rituximab, cyclophosphamide, doxorubicin, vincristine, and prednisolone (R-CHOP) and high-dose methotrexate for DLBCL, the patient is demonstrating an asymptomatic, benign course with reduced residual lesions. Her symptoms and coagulation abnormality with Trousseau syndrome improved simultaneously with tumor shrinkage (FDP 4.2 μg/mL, D-dimer 1.1 μg/mL, TAT 3.2 μg L). We are planning to initiate radiation therapy after the completion of four courses.

## Discussion

Skull tumors with aggressive bone destruction can arise from a variety of malignancies, including osteosarcoma, chondrosarcoma, chordoma, Langerhans cell histiocytosis, dermoid cyst, epidermoid cyst, meningioma, and plasmacytoma. The occurrence of DLBCL restricted to the cranial vault is rare, although there are some reports of such disorders [6–8]. Nevertheless, we did not come across any report of primary DLBCL of the cranial vault complicated with Trousseau syndrome in our research.

In general, Trousseau syndrome means “hypercoagulable state associated with malignant tumor or disseminated intravascular coagulation and its accompanying migratory venous or arterial thromboembolism,” but there is no clear diagnostic standard [9]. In the present case, despite the absence of arrhythmia, cardiac disease, collagen disease, and arteriosclerotic lesions, we found coagulation abnormalities and multiple cerebral infarctions in both cerebral hemispheres. Since there was an osteoclastic neoplastic lesion in the skull, we considered Trousseau syndrome with malignant tumor. Although most malignant tumors as a cause of Trousseau syndrome are solid cancers, often identified as gynecologic tumors, the frequency varies depending on reports [10]. Histologically, it often occurs as adenocarcinoma, particularly mucin-producing adenocarcinoma [11], and colorectal cancer has a high incidence (2–15.9%) among malignant tumors [5]. Lymphoma is recognized as a risk factor for Trousseau syndrome by the Khorana score, an observation supported by other research, but they consider blood cell abnormalities and chemotherapy due to lymphoma as a cause of Trousseau syndrome [12, 13]. Lymphoma with no treatment and no blood cell abnormality, as in this case, is unlikely to be considered as a risk of Trousseau syndrome. Chemotherapy for DLBCL simultaneously reduced tumor size and improved the coagulation abnormality with no recurrence of cerebral infarction. Successful treatment of the primary disease thereby improved symptoms of Trousseau syndrome in the present case, similar to what has been observed for other cases [1, 14].

In previous reports, primary DLBCL of the cranial vault was often considered as osteoclastic lesion [15], and intracranial symptoms such as seizure and hemiplegia were reported in some cases [4, 16, 17]. Although there were no intracranial symptoms and no development to the dura mater or the brain in the present case, it was similar to the past cases with regard to the extensive skull destruction. However, it is said that metastatic skull tumors may often have a similar osteoclastic nature and could be very difficult to differentiate from vault lymphoma [18]. According to a report by Uemura *et al*. comparing vault lymphoma with other skull tumors with regard to osteoclastic patterns, bone destruction of vault lymphoma developed diffusely and gradually, but cortical bone was relatively easy to be maintained and could show uneven bone destruction [19]. In the present case, bone destruction observed on CT scans and in surgical findings was localized in comparison with the lesion area imaged by CEMRI. On the other hand, metastatic skull tumor would have developed to destroy the entire bone structure from the early stage [19].

## Conclusion

As skull tumors can result from various malignancies, they may often be difficult to diagnose. Our observations in this case suggest that Trousseau syndrome may develop in association with DLBCL. Furthermore, DLBCL should be considered as a differential diagnosis for all lesions of the cranial vault that lack invasion of the central nervous system.

## Data Availability

We make our references in the manuscript available for testing by reviewers.
